# SUMO‐modified insulin‐like growth factor 1 receptor (IGF‐1R) increases cell cycle progression and cell proliferation

**DOI:** 10.1002/jcp.25818

**Published:** 2017-04-25

**Authors:** Yingbo Lin, Hongyu Liu, Ahmed Waraky, Felix Haglund, Prasoon Agarwal, Helena Jernberg‐Wiklund, Dudi Warsito, Olle Larsson

**Affiliations:** ^1^ Department of Oncology and Pathology CCK R8: 04 Karolinska Institutet Stockholm Sweden; ^2^ Laboratory of Aquatic Animal Nutrition Feed Fisheries College Guangdong Ocean University Zhanjiang China; ^3^ Department of Immunology, Genetics and Pathology Rudbeck Laboratory Uppsala Sweden; ^4^ Department of Laboratory Medicine (LABMED) H5 Division of Clinical Immunology Karolinska University Hospital Stockholm Sweden

**Keywords:** cancer, cell cycle, IGF‐1R, proliferation, SUMOylation

## Abstract

Increasing number of studies have shown nuclear localization of the insulin‐like growth factor 1 receptor (nIGF‐1R) in tumor cells and its links to adverse clinical outcome in various cancers. Any obvious cell physiological roles of nIGF‐1R have, however, still not been disclosed. Previously, we reported that IGF‐1R translocates to cell nucleus and modulates gene expression by binding to enhancers, provided that the receptor is SUMOylated. In this study, we constructed stable transfectants of wild type *IGF1R (WT)* and triple‐SUMO‐site‐mutated *IGF1R (TSM)* using *igf1r* knockout mouse fibroblasts (R‐). Cell clones (R‐WT and R‐TSM) expressing equal amounts of IGF‐1R were selected for experiments. Phosphorylation of IGF‐1R, Akt, and Erk upon IGF‐1 stimulation was equal in R‐WT and R‐TSM. WT was confirmed to enter nuclei. TSM did also undergo nuclear translocation, although to a lesser extent. This may be explained by that TSM heterodimerizes with insulin receptor, which is known to translocate to cell nuclei. R‐WT proliferated substantially faster than R‐TSM, which did not differ significantly from the empty vector control. Upon IGF‐1 stimulation G1‐S‐phase progression of R‐WT increased from 12 to 38%, compared to 13 to 20% of R‐TSM. The G1‐S progression of R‐WT correlated with increased expression of cyclin D1, A, and CDK2, as well as downregulation of p27. This suggests that SUMO‐IGF‐1R affects upstream mechanisms that control and coordinate expression of cell cycle regulators. Further studies to identify such SUMO‐IGF‐1R dependent mechanisms seem important.

## 1 INTRODUCTION

The insulin‐like growth factor receptor 1 (IGF‐1R) is a receptor tyrosine kinase with pivotal roles in the physiological regulation of growth during fetal and adult life (Perrini et al., 2010). Ligand activation of cell membranous IGF‐1R induces activation of several downstream signaling pathways, for example, the PI3K/AKT and the MAPK/ERK pathways (Laviola, Natalicchio, & Giorgino, 2007). IGF‐1R signaling has been reported to promote cell proliferation, survival, and hypertrophy, and is strongly implicated in the development and progression of human cancer (Clemmons, 2007). Several types of cancer cells are heavily dependent on IGF‐1R for survival, which has been demonstrated both in vivo and in vitro (Baserga, 2009; Resnicoff et al., 1996). However, clinical trials with anti‐IGF‐1R therapy have yielded disappointing results due to toxicity or poor tumor response (Beckwith & Yee, 2015; Pappo et al., 2014). This has prompted further investigation of IGF‐1R signaling and functionality.

Recently, we showed that the IGF‐1R undergoes SUMOylation, which leads to nuclear translocation and gene activation through binding to enhancers or nuclear proteins (Packham et al., 2015; Sehat et al., 2010; Warsito, Lin, Gnirck, Sehat, & Larsson, 2016; Warsito, Sjöström, Andersson, Larsson, & Sehat, 2012). Introduction of site specific mutations corresponding to three evolutionary conserved SUMOylation sites (Lys1025, Lys1100, and Lys1120) in *IGF1R* decreased nuclear IGF‐1R (nIGF‐1R) and abolished its gene regulatory effects while retaining IGF‐1R kinase‐dependent signaling (Sehat et al., 2010).

Since our discovery and first characterization of nIGF‐1R, several new aspects on it have been reported. nIGF‐1R has been linked to adverse patient outcome or tumor progression in renal cell carcinomas, embryonal rhabdomyosarcomas, and synovial sarcomas (Aleksic et al., 2010; Palmerini et al., 2015; van Gaal et al., 2013). It has been proposed as a marker of overall survival and progression‐free survival in patients with soft tissue sarcomas and osteosarcomas treated with anti‐IGF‐1R antibody therapy (Asmane et al., 2012). High levels of nIGF‐1R has also been reported in several cancer cell lines, including human alveolar rhabdomyosarcoma, hepatocellular, prostate, and breast carcinoma, as well as acute myeloid leukemia cells (Aslam et al., 2013; Chein, Kuo, Liao, Wang, & Yu, 2016; Deng et al., 2011; Sarfstein et al., 2012; Zhang et al., 2015).

As SUMO1 modification is critical for IGF‐1R's transactivating effects and nuclear receptor is linked to adverse clinical outcome and tumor biological properties, we, here, sought to investigate whether the SUMOylation status of IGF1R may affect cell proliferation. For this purpose, we established a model system using *igf1r−*/− knockout murine embryonic fibroblast transfected with either wild type *IGF1R* or *IGF1R* with mutated SUMOylation sites.

## 2 MATERIAL AND METHODS

### 2.1 Cell lines and reagents

The *igf1r* deficient R‐cell line was isolated from mouse embryos with a targeted disruption of the *igf1r* gene by Dr. Renato Baserga's group (Sell et al., 1993). pBABE‐Puro retroviral expression vector (#RTV‐001‐PURO) and Platium‐E packaging cell line (#RV‐101) were bought from Cell Biolabs Inc. (San Diego, CA).

Agar (#214220), BrdU (#550891), 7‐AAD (#559925), mouse anti‐IGF1R (#556000), and FITC labeled mouse anti‐BrdU (#347583) was purchased from Becton, Dickinson and Company (San Jose, CA). Polybrene (sc‐134220), antibodies against GAPDH (sc‐25778), cyclin A (SC‐751), cyclin D (SC‐450), cyclin E (SC‐247), CDK2 (SC‐748), and normal mouse IgG (sc‐2025) were obtained from Santa Cruz Biotechnology Inc. (Santa Cruz, CA). IGF‐1R (#3027), pAkt (#9275), pErk (#9101), SUMO‐1 (#4940), CDK4 (#2906), p27 kip1 (#3698), InsR β (#3020), and phospho‐tyrosine (#9411) antibodies were purchased from Cell Signaling Technology (Danvers, MA). Cyclin B1 (ab181593) and CDK1 (ab18) antibodies were provided by Abcam (Cambridge, MA). qRT‐PCR primers for IGF1R (#Hs00609566_m1) and GAPDH (#Mm99999915_g1), puromycin (#A11138), and protein G Dynabeads (#10004D) were provided by Life Technologies (Carlsbad, CA).

### 2.2 Retrovirus production

Wild type (WT) and triple‐SUMO1‐site‐mutated (TSM) *IGF1R* expression sequences were PCR amplified from vectors previously generated in our group (Sehat et al., 2010) and sub‐cloned into pBABE‐puro vector. After sequencing confirmation, the pBABE‐puro, pBABE‐WT, and pBABE‐TSM vectors were transfected into Platium‐E cell line respectively. At 48 and 72 hr post transfection, the supernatants with packaged retrovirus particles were collected and filtered through 0.45 μm polysulfonic filters before infecting R‐cells.

### 2.3 Knock‐in of *WT/TSM‐IGF1RWT/TSM‐IGF1R*

The R cells were seeded in 25 cm^2^ flasks at 30% confluency in complete DMEM medium. For infection, 5 ml retrovirus supernatants with 8 μg/ml polybrene were added to each flask at 24, 48, and 72 hr post seeding. pBABE‐puro, pBABE‐WT, and pBABE‐TSM retrovirus particles were employed for mock, WT‐IGF1R, and TSM‐IGF1R transfection, respectively. Four days after seeding, 1 μg/ml puromycin was supplemented in to the culture medium to eliminate untransfected cells. Mediums were changed every third day until the cells were 90% confluent.

### 2.4 Generation of monoclonal cell lines

Transfected R‐cells were diluted to 10 viable cells per ml medium and 0.1 ml was dispensed to each well in 96‐well cell culture plates. After 10 days’ incubation at 37°C, wells with single clone were isolated and expanded. qRT‐PCR was applied to determine the relative *IGF1R* expression level in each clone, using a delta‐delta Ct protocol and *GAPDH* as endogenous control.

### 2.5 Immunoprecipitation (IP)

For each cell line, 10^7^ cells were harvested and boiled in 100 µl TSD buffer (50 mM Tris‐Cl, 1% SDS, 5 mM DTT, 20 mM N‐Ethylmaleimide, and 1× protease and phosphatase inhibitor) for 10 min, followed by brief sonication and centrifugation at 16,000 g for 10 min. The supernatants were diluted with 1.2 ml of TNN buffer (50 mM Tris‐Cl, 250 mM NaCl, 5 mM EDTA, 0.5% NP‐40, 20 mM N‐Ethylmaleimide, and 1×protease and phosphatase inhibitor). IGF‐1R was pull down with 5 µl mouse anti‐IGF1R antibody and 10 µl protein G Dynabeads overnight at 4°C. Precipitated proteins were separated by SDS‐PAGE, transferred onto a nitrocellulose membrane and blotted with specific antibodies.

### 2.6 XTT cell proliferation assay

In a 96‐well plate, 3 × 10^3^ of R‐puro, R‐WT, R‐TSM, WT‐2D5, or TSM‐3B4 cells were evenly seeded in complete medium. Cell proliferation was monitored every 24 hr with the Cell Proliferation Kit II (Cat. No. 11465015001, Roche, Basel, Switzerland) following the manufacture's instruction. Five replicates were included for each time point.

### 2.7 Cell cycle distribution and apoptosis analysis

R‐puro, R‐WT, and R‐TSM cells were seeded in 6 cm dishes at 70% confluency and starved for 36 hr before stimulated with 50 ng/ml IGF‐1 ligand. At 1 hr before harvest, 10 μM BrdU was added to the culture medium. At specific time points (0, 10, 16, and 24 hr) post stimulation, the cells were harvested and fixed in 70% Ethanol at −20°C overnight. Immunostaining was carried out with anti‐BrdU following the manufacturer's instruction. A total of 50 µg/ml 7‐amino actinomycin D (7‐AAD) was used to stain the DNA. Cells in G0/G1, S, and G2/M phases were gated using an ACEA NovoCyte™ 3000 flow cytometry with the NovoExpress™ software based on their BrdU and 7‐AAD content. R‐puro, R‐WT, or R‐TSM cells were cultured under basal condition at 70% confluency. Apoptosis was studied using Annexin V/PI method (Annexin V‐FLUOS staining kit, Roche, Mannheim, Germany) according to the manufacturer's protocol. Flow cytometric analysis was immediately performed using the NovoCyte™ 3000.

### 2.8 Soft agar colony formation assay

Six‐well plates were precast with 2 ml DMEM medium supplemented with 10% FBS and 0.5% agar as the bottom layer. R‐puro, R‐WT, and R‐TSM cells were trypsinized, counted, and suspended in basal DMEM medium with 0.3% agar at a concentration of 500 cells per milliliter. Two milliliters of the cell solutions were plated onto the solidified bottom layer in each well, and cultured at 37°C. A volume of 500 µl basal DMEM medium was added to each well every 4 days without disturbing the agar layers. After 14 days, the colonies in each well were stained with 200 µl of nitroblue tetrazolium chloride solution overnight at 37°C and counted. Five replicates were carried out for each cell line.

### 2.9 DuoLink in situ proximity ligase assay (PLA)

Antibodies against IGF‐1Rβ and insulin receptor β were used to detect the colocalization of IGF‐1R and insulin receptor in R‐puro, R‐WT, and R‐TSM cells according to the manufacturer's instructions. IGF1R was simultaneously stained with green Alexa Fluor® 488 conjugate secondary antibody. Cell nuclei were visualized by DAPI counter staining. Images were acquired with a Zeiss (Oberkochen, Germany) Axioplan2 imaging microscope at 40× magnification and analyzed in AxioVision 3.1 software.

## 3 RESULTS

### 3.1 Cell line verification and characterization

**Figure 1:**
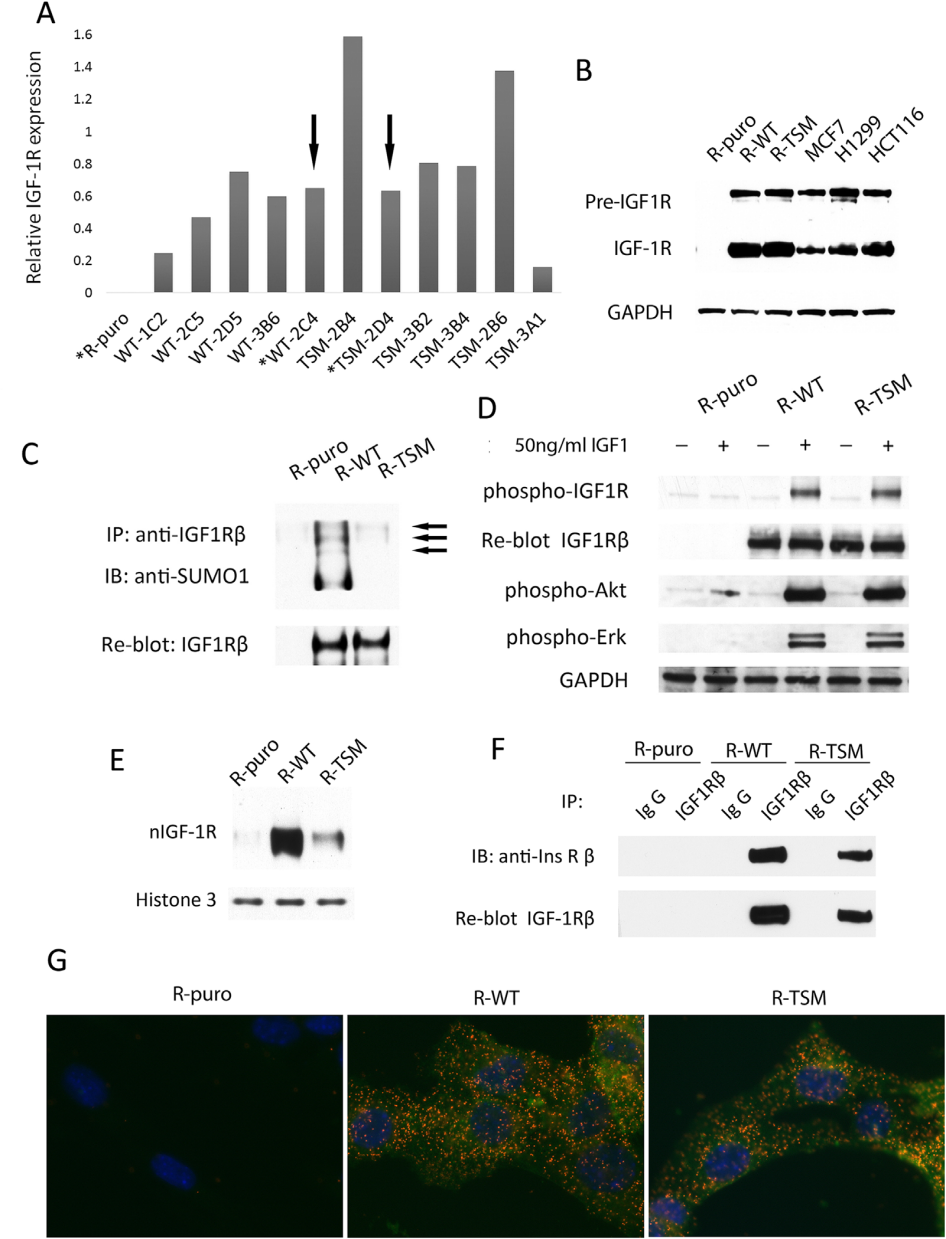
Characterization of R‐cells (*igf1r−*/−) stably expressing wild type (WT) or triple SUMO‐sites mutated (TSM) *IGF1R*. (A) Relative *IFG1R* mRNA transcription levels in selected transfected R‐cell clones were determined by qRT‐PCR. Two cell lines with equal expression of WT (WT‐2C4) and TSM (TSM‐2D4) *IFG1R* mRNA (marked with arrows) were chosen for further investigation and named R‐WT and R‐TSM, respectively. R‐puro was an empty vector control. (B) IGF‐1R and pre‐IGF1R protein expression in R‐puro, R‐WT, R‐TSM cells compared to the cancer cell lines MCF7 (breast cancer), H1299 (lung cancer), HCT116 (colon cancer) were determined by immunoblotting (IB) with anti‐IGF‐1Rβ. GAPDH was blotted as loading control. (C) SUMOylation of IGF‐1R in R‐puro, R‐WT, and R‐TSM cell lines were determined by immunoprecipitation (IP) of IGF‐1R and IB for SUMO1. The three predicted SUMO‐IGF‐1R bands are indicated by arrows. Re‐blot of IGF‐1Rβ as an input IP control. (D) IGF‐1R tyrosine kinase activity was assessed by receptor and substrate phosphorylation. IGF‐1R was IPed from lysates from R‐puro, R‐WT, and R‐TSM cells that had been subjected to 36‐h serum starvation with or without subsequent 10‐min IGF‐1 stimulation, and blotted with anti‐phospho‐tyrosine antibody. Re‐blot of IGF‐1R β served as input control. The lysates were also directly blotted for phosphorylated Erk (pErk) and phosphorylated Akt (pAkt). GAPDH was used as loading control. (E) R‐puro, R‐WT, and R‐TSM cells were fractionized and the nucleus portions were immunoblotted with anti‐IGF1Rβ. Histone 3 was blotted as loading control. (F) Association of IGF‐1R and InsR was investigated by co‐IP. IGF‐1R IPs from R‐puro, R‐WT, and R‐TSM cell lysates were blotted for InsRβ. Re‐blot of IGF‐1Rβ served as control of IP. (G) Co‐localizations between IGF‐1R and InsR were visualized by PLA (red dots) in R‐puro, R‐WT, and R‐TSM cells. IGF‐1R and cell nuclei were counterstained with Alexa Fluor® 488 (Green) and DAPI (Blue), respectively. Data are representative of 3–5 experiments

The different *igf1r−*/− knockout cell clones transfected with WT or TSM *IGF1R* expressed variable mRNA levels of *IGF1R* as determined by qRT‐PCR (Fig. 1A). WT‐2C4 and TSM‐2D4 clones exhibiting equal *IGF1R* mRNA (Fig. 1A) and protein levels (Fig. 1B) were selected for further experiments and were named R‐WT and R‐TSM, respectively. The protein levels of *IGF1R* in R‐WT and R‐TSM were essentially in the range of those in common cancer cell lines (Fig. 1B). R‐puro (empty vector transfectant) showed no *IGF1R* mRNA or IGF‐1R expression (Fig. 1A and B).

SUMOylation of the transfected cell lines was investigated by immunoprecipitation of IGF‐1R followed by detection of SUMO1 by immunoblotting. We observed that SUMO‐modified IGF‐1R was restricted to R‐ WT cells (Fig. 1C).

To compare the activity of IGF‐1R signaling in the transfected cell lines, phosphorylation of IGF‐1R, Akt, and Erk was determined before and after stimulation with 50 ng/ml IGF‐1 in serum‐starved cells. Whereas R‐puro showed no response (except a faint increase in pAkt that is judged as unspecific) to ligand stimulation, both R‐WT and R‐TSM showed clear and equal phosphorylation of IGF‐1R, Akt, and Erk (Fig. 1D). This supports that TSM modified IGF‐1R has an intact tyrosine kinase activity.

Next, the cell lines were subjected to nuclear extraction. Nuclear TSM‐IGF‐1R was detectable, but at a much lower level compared to WT‐IGF‐1R (Fig. 1E). A possible explanation could be heterodimerization of IGF‐1R with insulin receptor (InsR). Accordingly, InsRβ co‐precipitated with IGF‐1Rβ in both R‐WT and R‐TSM cell lines (Fig. 1F). This was confirmed by PLA, which indicated the co‐localization of InsRβ and IGF‐1Rβ in all compartments of the cells, including nuclei (Fig. 1G).

### 3.2 SUMOylated IGF‐1R increases proliferation in R‐cells

**Figure 2:**
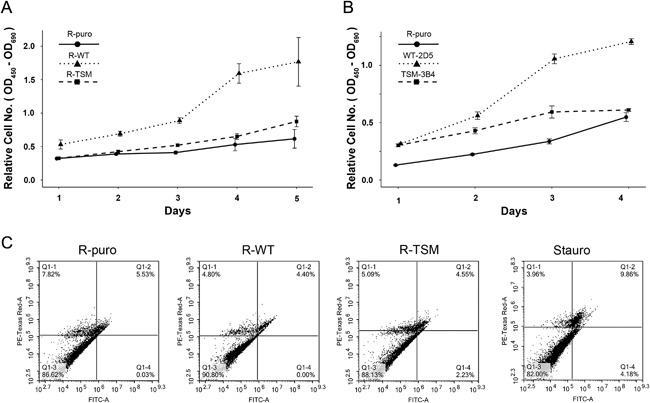
Comparison of cell proliferation and apoptotic cell death. (A) Equal numbers of R‐puro, R‐WT, or R‐TSM were seeded in 96‐well plates and cultured under basal condition. Proliferation of cells were monitored by XTT proliferation assay kit every 24 hr. Each time‐point represents the average (*n* = 5) proliferation, with whiskers representing a 95% confidence interval. (B) R‐puro, WT‐2D5, or TSM‐3B4 cells were investigated for proliferation exactly as described in A. (C) Apoptosis/cell death in R‐puro, R‐WT, or R‐TSM cells under basal condition was assessed using Annexin V (X‐axis)/PI (Y‐axis) protocol. A total of 1 μM (final concentration) staurosporine was used as positive control. Data are representative of three experiments

Cell proliferation was measured daily over five consecutive days using an XTT colorimetric assay. During the whole experimental time R‐WT showed a significantly higher proliferation (*t*‐test, *p *< 0.05 for all time points) than both R‐puro and R‐TSM (Fig. 2A). At the final measure (day 5), R‐WT had a 3.6‐fold increase in viable cells as compared to R‐puro (2.1‐fold increase) and R‐TSM (2.7‐fold increase). R‐TSM showed only a minor increase in proliferation as compared to R‐puro (significantly higher at days 3 and 5). To verify these results, another set of stable cell clones with equal expression of IGF‐1R, WT‐2D5, and TSM‐3B4 (cf. Fig. 1A), was compared over a 4‐day culturing. Similar to R‐WT, WT‐2D5 showed a significantly higher proliferation (*p *< 0.05) than both TSM‐3B4 and R‐puro (Fig. 2B).

The apoptotic/cell death rate of the cell lines was assessed by FACS after staining with Annexin V/PI, for detection of early and late apoptosis. As shown in Fig. 2C, the relative numbers of apoptotic cells in the three cell lines were essentially comparable. Staurosporin was used as a positive control.

### 3.3 SUMO modification of IGF‐1R increases G1‐S progression

**Figure 3:**
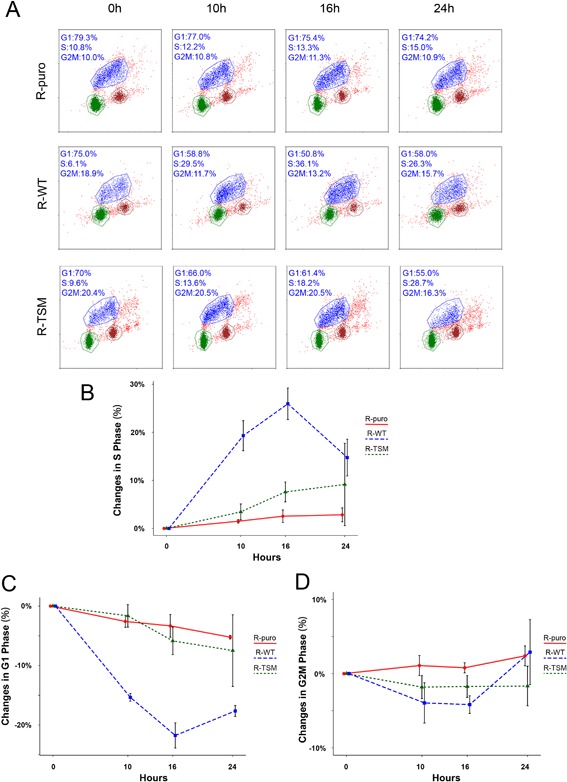
Comparison of ligand stimulated cell cycle progression. (A) R‐puro, R‐WT, and R‐TSM were synchronized by 36 hr serum starvation and then exposed to 50 ng/ml IGF‐1 for 0, 10, 16, and 24 hr. FACS analyses after double staining with 7‐amino actinomycin D (X‐axis) and BrdU (Y‐axis) are shown. (B–D) Percentage of changes of number of cells in S‐ (B), G1‐ (C), and G2M‐phase (D) upon IGF‐1 stimulation for 10, 16, and 24 hr as compared to unstimulated cells (0 hr). Experiments on R‐puro, R‐WT, and R‐TSM were performed as described in A. Results represent means of three independent experiments. Whiskers represent 95% confidence intervals

Based on the results on cell proliferation, we investigated the effects of SUMOylation status on cycle progression. The cells lines were synchronized by serum starvation for 36 hr to accumulate most of the cells in G1‐phase. After 0, 10, 16, or 24 hr of IGF‐1 stimulation the percentages of cells in different cell cycle phases were determined using BrdU/PI double staining, followed by FACS (Fig. 3A). As shown in Fig. 3B and C, the most substantial changes observed were the increase in S‐phase and corresponding decrease in G1‐phase in ligand stimulated R‐WT. After a 26% increase at 16 hr, the number of S‐phase cells was clearly decreased by 24 hr. The corresponding response in R‐TSM was much weaker with highest increase (8%) at 24 hr. No significant changes were detected in R‐puro cell line (Fig. 3B and C). During the 24 hr experiment, we could not detect any significant changes in G2/M phase in any of the cell lines, although R‐WT exhibited a trend of increased G2/M (Fig. 3D). Probably a substantial portion of IGF‐1 stimulated W‐RT cells had undergone mitosis before 24 hr, which is in line with the increase in G1 cells at 24 hr (Fig. 3C).

**Figure 4:**
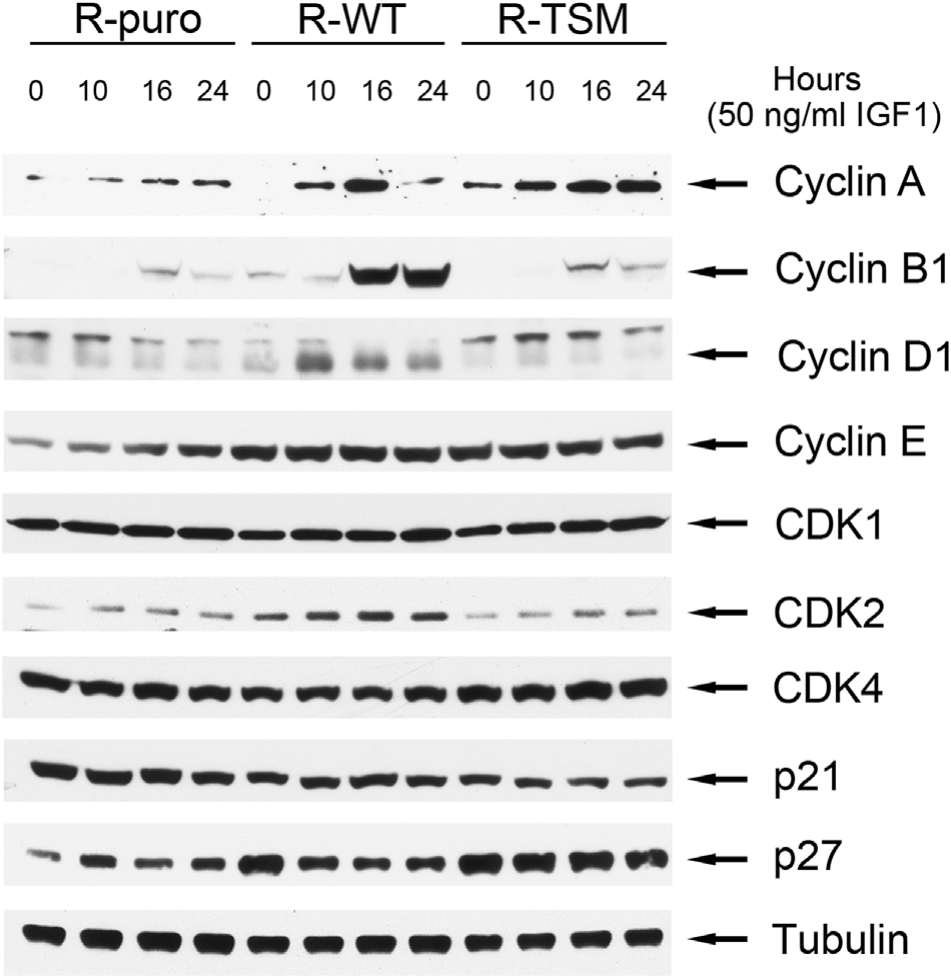
Comparison of expression of cell cycle proteins. R‐puro, R‐WT, and R‐TSM were synchronized by 36 hr serum starvation and then exposed to 50 ng/ml IGF‐1 for 0, 10, 16, and 24 hr. Protein expression of cyclin A, cyclin B1, cyclin D1, cyclin E, CDK1, CDK2, CDK4, p21, and p27 was detected by immunoblotting. Tubulin was blotted as loading control. Data are representative of >3 experiments

To investigate whether the cell cycle alterations correlate with changes in the expression of cell cycle proteins, the expression of main cyclins, CDKs, and CIP/KIP CDK inhibitors, regulating different stages of the cell cycle, were analyzed. The most prominent changes occurred in IGF‐1 treated R‐WT cells. An increase occurred in expression of the G1/S phase cyclin D1 after 10 hr, which was followed by increase in S phase cyclin A and G2/M cyclin B1 after 16 and 24 hr, respectively (Fig. 4). These changes match the cell cycle kinetic events (Fig. 3). The enhanced expression of cyclin B1 together with the trend for increase in G2/M and G1 at 24 hr suggests that S‐G2 progression is fastened in ligand stimulated R‐WT cells (Figs., 3 4). No detectable changes in cyclin E expression were seen (Fig. 4). Compared to R‐WT, ligand stimulated R‐TMS showed weaker increase in cyclin A and B1, and cyclin D1 was only hardly affected (Fig. 4). The S‐phase CDK2 was the only CDK exhibiting a detectable upregulation upon IGF‐1 stimulation during the experimental time and it was strongest for R‐WT (Fig. 4). Upon ligand treatment of R‐WT, the expression of CIP/KIP p27 was decreased, while it was not affected in R‐TSM. No detectable changes in p21 were seen in any of the cell lines (Fig. 4). Except a small increase in cyclin B1, no changes were observed in R‐puro.

Taken together, the cell cycle kinetic data (Fig. 3) and changes in cell cycle proteins (Fig. 4) suggest that SUMO modification mainly regulates IGF‐1R‐dependent cell proliferation through increasing G1‐S progression. As several cell cycle proteins were affected, their expression is most likely conducted by upstream events.

### 3.4 SUMOylated IGF‐1R increases anchorage independent cell growth

**Figure 5:**
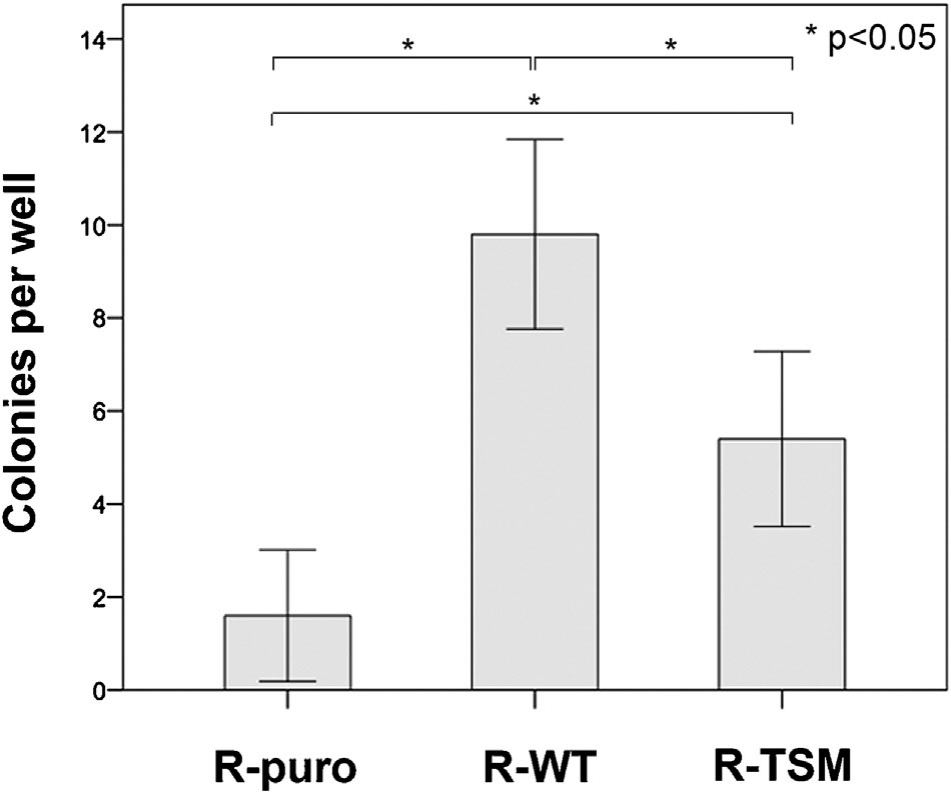
Comparison of colony formation. Soft agar colony formation assay on R‐puro, R‐WT, and R‐TSM was performed as described in Materials and Methods. Experiments were stopped after 14 days for counting of colonies. Each column represents the mean number of colonies formed per well (total 5‐wells per cell line and experiment). Whiskers represent 95% confidence intervals. *p*‐values are indicated. Data are representative of three experiments

To investigate the potential role of SUMOylated IGF‐1R in anchorage independent growth, the cell lines were analyzed for colony formation using soft agar assay (Fig. 5). Cells were cultured in soft agar for 2 weeks. The R‐WT cell line formed significantly more colonies than both R‐puro and R‐TSM (*p *< 0.05). Significantly increased colony formation was also seen in R‐TSM (*p *< 0.05) compared to R‐puro. Thus, IGF‐1R's canonical signaling seems also important for anchorage independent growth.

## 4 DISCUSSION

Nuclear localization of IGF‐1R is emerging as a potentially important factor in tumor pathophysiology and clinical prediction. However, its specific role in a cell physiological context is still poorly understood. Initially, we reported that SUMOylation is important for nuclear IGF‐1R‐induced transactivation (Sehat et al., 2010). In this study, we established a cell model (R‐WT/R‐TSM) for assessment of cellular responses in cells expressing SUMO‐modified IGF‐1R versus non‐SUMO‐modified IGF‐1R. We focused on potential effects on cell proliferation and cell cycle progression.

We demonstrated that R‐WT cells (expressing SUMO‐modified receptor) were coupled to a significant increase in cell proliferation mainly through G1‐S phase transition as compared to R‐TSM cells (expressing non‐SUMO‐modified receptor). These data provide direct evidence that SUMOylation is important for IGF‐1R‐induced cell proliferation. In consistence with our finding, Zhang et al. (2015) showed that co‐expression of IGF‐1R containing SUMOylation site mutations (i.e., K1025R and K1100R) substantially decreased proliferation in acute myeloid leukemia cells. Furthermore, a correlation between estrogen receptor positive breast cancer cells and SUMOylated IGF‐1R in cell nuclei was demonstrated by Sarfstein et al. (2012).

TSM‐mutated and wild‐type IGF‐1R had intact and equal kinase activity as determined by Akt and Erk phosphorylation after IGF1‐stimulation. We were, therefore, surprised to observe that R‐TSM did not differ much in proliferation and cell cycle progression compared to R‐puro (mock transfected cells), suggesting a limited proliferative effect of membranous IGF‐1R in this model. Some cell types have previously been described to respond with differentiation rather than proliferation upon IGF‐1R stimulation (Baserga, 2009). We could confirm that the difference in proliferation rate was not due to difference in rate of apoptotic cell death. While an even starker effect was seen in WT transfected cells, we observed a clear effect of TSM transfection on soft agar colony formation.

Hypothetically, the canonical IGF‐1R signaling may affect cell growth predominantly through increasing cell survival (e.g., Baserga, 2009; Resnicoff et al., 1996), whereas SUMO‐dependent signaling mainly affects cell proliferation. The finding that co‐expression of IGF‐1R^K1025R/ K1100R^ in leukemia cells decreased proliferation but did not cause apoptosis (Zhang et al., 2015) supports this hypothesis. Under the experimental conditions (basal) applied in the present study, we could not detect any difference in rate of apoptotic cell death in R‐WT and R‐TSM.

The observation that SUMOylation of IGF‐1R is important for cell growth may partly explain the more aggressive phenotype of tumors expressing nuclear IGF‐1R. SUMOylated IGF‐1R might even be a more accurate biomarker than nuclear IGF‐1R in clinical management. For example, in situ proximity ligation assay (PLA) for detection of SUMO‐IGF‐1R in tumor specimen may become a feasible method in this respect.

The mechanism underlying the ability of SUMOylated IGF‐1R to induce proliferation and cell cycle progression remain requires further studies. In the current study, we found increased expression of cyclin D1, cyclin A, and CDK2 as well as decrease in p27 in R‐WT cells, events that are known to drive cells from G1‐ to and through the S‐phase. As several cell cycle regulators are involved, upstream mechanisms most likely control and coordinate their expression. Such mechanisms might be dependent on the SUMOylation status of IGF‐1R. We, previously, showed that nIGF‐1R binds to enhancer‐like regions and that WT‐IGF‐1R increased reporter gene activity if these regions were inserted in the reporter vector (Sehat et al., 2010). In contrast, TSM‐IGF‐1R inhibited the gene activity. These data suggest that SUMOylation is important for nIGF‐1R‐induced transactivation. This could depend on that SUMO‐ modification regulates nuclear import of IGF‐1R but also that SUMO1‐IGF‐1R may directly influence the transactivation process, for example, by binding to enhancers (Sehat et al., 2010) or to transcription factors (Warsito et al., 2012) or by interfering with epigenetic mechanisms (Warsito et al., 2016).

In the present study, we show that TSM‐IGF‐1R also can translocate to the cell nucleus, but to a much lower extent compared to WT‐IGF‐1R. This suggests that SUMOylation of IGF‐1R is not an absolute requirement for nuclear translocation. Alternatively, TSM could be transported to the cell nucleus through heterodimerizing with the InsR. Actually, we could show that IGF‐1R co‐localizes with InsR in MEFs. Irrespective of which, the biological response (i.e., cell proliferation) is dependent on the SUMOylation status of IGF‐1R.

**Figure 6:**
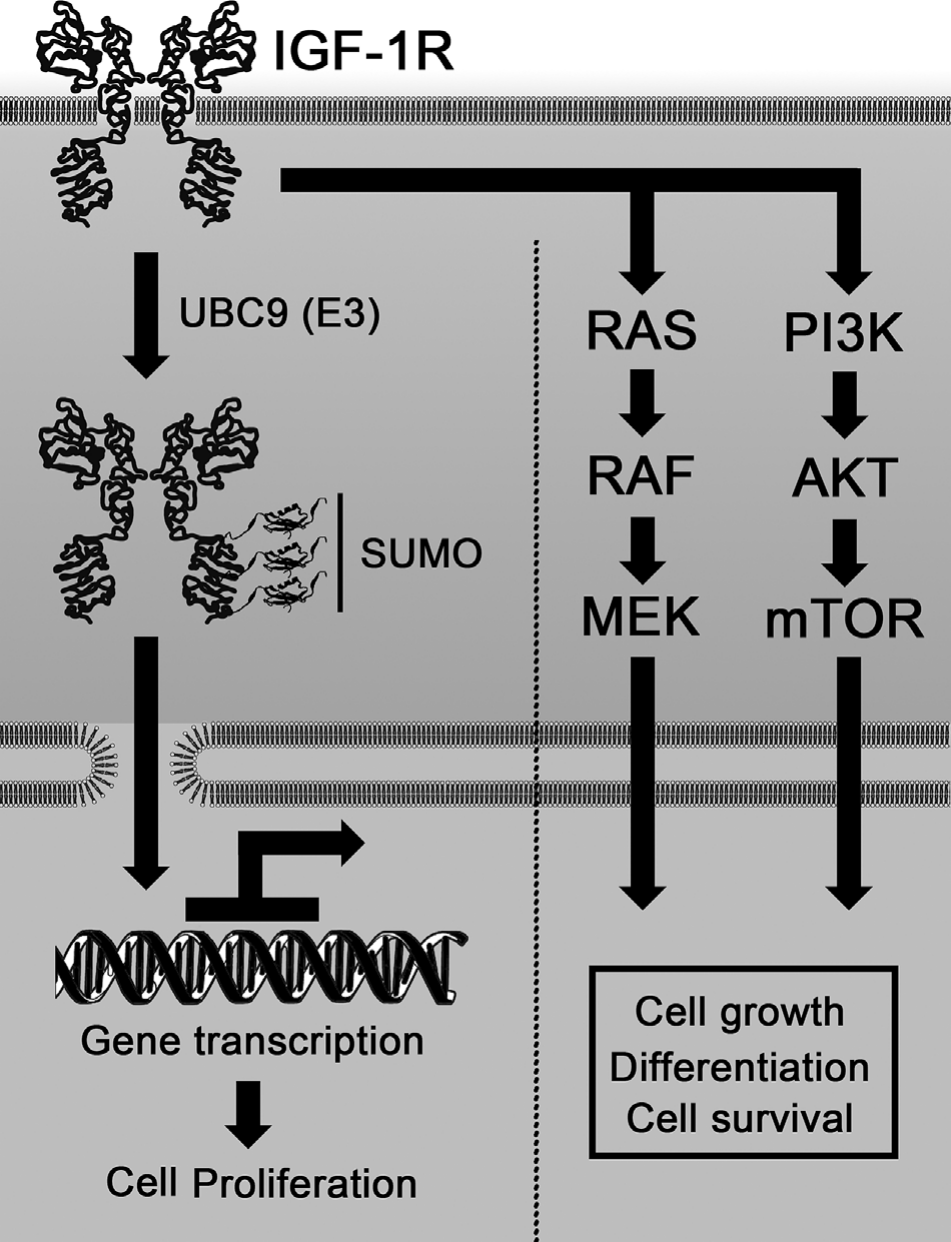
Schematic model of cellular responses of IGF‐1R. Besides traditional signaling pathways, the SUMO‐modification of IGF‐1R may modulate cell proliferation through gene transactivation. The model represents a hypothesis based on present and previous results

Based on the present and previous findings, we hypothesize that SUMO‐dependent IGF‐1R‐induced gene activation may explain the effects on cell proliferation. Accordingly, the remaining step is to identify the specific genes involved in this context. For this purpose, our model system, R‐WT versus. R‐TSM, may be helpful. Applying expression microarray or RNA sequencing, it should be possible to identify genes whose expression differs between R‐WT and R‐TSM. Some of these genes may, for example, control and coordinate expression of cell cycle proteins (like cyclin D1, A, and CDK2), but apart from genes regulating cell proliferation the model system may also identify SUMO‐IGF‐1R dependent gene expressions connected to other cellular functions (like apoptosis and cellular migration). Figure 6 briefly summarizes our hypothesis.
